# Novel Genome Sequences of Ophidiomyces ophidiicola, the Causative Agent of Snake Fungal Disease

**DOI:** 10.1128/mra.00093-23

**Published:** 2023-06-26

**Authors:** Erin B. Brosnan, Jenna N. Palmisano, Julianna K. Martin, Robert R. Fitak

**Affiliations:** a Department of Biology, University of Central Florida, Orlando, Florida, USA; University of Maryland School of Medicine

## Abstract

Ophidiomyces ophidiicola is a globally distributed fungal pathogen of snakes. This study reports genome assemblies for three novel isolates that were derived from hosts originating in the United States, Germany, and Canada. The assemblies have a mean length of 21.4 Mbp, with coverage of 116.7×, and will contribute to wildlife disease research.

## ANNOUNCEMENT

The fungus Ophidiomyces ophidiicola of the order Onygenales is an emerging infectious pathogen that causes snake fungal disease in over 60 snake species globally (1). Snake fungal disease, or ophidiomycosis, is initially a skin infection that can result in severe lesions, scale abnormalities, and granulomas (2). Ophidiomycosis can disrupt the reproductive schedules of snakes, as well as their foraging and thermoregulatory behaviors (3). O. ophidiicola is widespread in native snake species of the eastern United States and has shown high mortality rates in some species under both field and experimental conditions (e.g., massasauga rattlesnakes [Sistrurus catenatus]) (4, 5). Recent phylogenetic analysis characterized 82 independent isolates of O. ophidiicola into three clades, with isolates from wild-caught snakes of North America forming a monophyletic clade that is highly divergent from the others, including isolates from Europe (6). Additional genomic resources and assessments are needed to understand the origin and distribution of O. ophidiicola and how its evolutionary history might have shaped pathogenicity and virulence. Here, we report genome assemblies from three new isolates of O. ophidiicola, which were obtained from the University of Alberta Microfungus Collection and Herbarium Centre for Global Microfungal Biodiversity (UAMH) (https://www.uamh.ca) and originated from an eastern foxsnake (Pantherophis gloydi) (UAMH11863) from Canada, a salt marsh snake (Nerodia clarkii) (UAMH10716) from the United States, and a garter snake (*Thamnophis* sp.) (UAMH9832) from Germany.

The isolates were originally obtained from the previously described snake hosts and deposited at the UAMH (Table 1) (7–9). The stored isolates were cultured on Sabouraud dextrose agar at 25°C for 7 to 14 days, followed by DNA extraction using the FastDNA SPIN kit for soil (MP Biomedicals LLC, Irvine, CA, USA). Paired-end, 2 × 150-bp libraries were prepared using the Illumina TruSeq DNA kit and sequenced on the Illumina NovaSeq 6000 system. Sequencing quality control and both nuclear and mitogenome assemblies were performed using the Automatic Assembly for the Fungi v0.3.3 pipeline (https://github.com/stajichlab/AAFTF) with the parameters –mincontiglen 200 –iterations 3 and otherwise default parameters. In short, the pipeline included read trimming and adapter removal with BBDuk v38.84.0 (http://sourceforge.net/projects/bbmap), mitochondrial genome assembly with NOVOPlasty v4.3.1 (10) using the mitogenome of O. ophidiicola strain CBS 122913 (GenBank accession number NW_026054640.1) as a seed, nuclear assembly with SPAdes v3.13.1 (11), contamination removal with NCBI VecScreen (https://www.ncbi.nlm.nih.gov/tools/vecscreen) and sourmash v3.1.0 (12), and three rounds of polishing with Pilon v1.24 (13). The nuclear assemblies are summarized in Table 1. Metrics of assembly lengths, *N*_50_ values, contig counts, and GC contents are consistent with current O. ophidiicola genomic sequences (6, 14). These genomic assemblies provide a valuable resource for future comparative genomic analyses, which are critical for the long-term management of ophidiomycosis and the prevention of potential catastrophic declines induced by this emerging pathogen.

**TABLE 1:** Sequencing metrics for the three Ophidiomyces ophidiicola isolates

Isolate	Original host isolation report	Total no. of raw reads	Total no. of filtered reads	Assembly length (bp)	No. of contigs	*N*_50_ (bp)	GC content (%)	Mean coverage (×)
UAMH9832	Vissiennon et al. (7)	16,360,218	15,203,428	21,569,881	361	365,001	47.80	91
UAMH10716	Sigler et al. (8)	20,162,182	18,656,940	21,414,035	344	516,346	47.88	120
UAMH11863	Davy et al. (9)	23,594,030	21,805,308	21,313,343	349	607,727	47.97	139

### Data availability.

This whole-genome project was deposited in DDBJ/ENA/GenBank under the accession numbers JAQGDZ000000000 (UAMH9832), JAQGEA000000000 (UAMH10716), and JAQGEB000000000 (UAMH11863). The mitogenomes for the isolates were deposited under accession numbers JAQGDZ010000362.1 (UAMH9832), JAQGEB010000350.1 (UAMH11863), and JAQGEA010000345.1 (UAMH10716); they are linked to BioProject PRJNA824508, with raw sequencing reads available under SRA accession numbers SRR21462230, SRR21462232, and SRR21462234, respectively. The versions described in this paper are the first versions.
